# Interconnected and resilient: A CGE analysis of AI‐driven cyberattacks in global trade

**DOI:** 10.1111/risa.14321

**Published:** 2024-06-04

**Authors:** Rehab Osman, Sherif El‐Gendy

**Affiliations:** ^1^ Oxford Brookes University Oxford UK; ^2^ School of Information Technology and Computer Science Nile University Cairo Egypt

**Keywords:** AI‐driven cyberattacks, CGE modeling, cyber resilience, international trade, supply chains

## Abstract

The burgeoning interconnectedness of global trade in the digital age not only presents enticing opportunities but also harbors potent vulnerabilities of artificial intelligence (AI)‐driven cyberattacks. This study explores the cascading impacts of these disruptive threats on economies, supply chains, and trade, utilizing the intricate lens of Computable General Equilibrium modeling.

Through meticulously designed simulation scenarios, we illuminate the potential economic ramifications of cyberattacks, with a focus on regions heavily reliant on digital technologies and interwoven supply chains. The analysis reveals significant declines in real GDP, trade prices and volumes, and trade route disruptions across regions. Notably, economies like China, the United States, the United Kingdom, and the EU, due to their deep integration in global networks, face pronounced vulnerabilities.

However, amidst this bleak landscape, hope emerges in the form of cyber resilience. The study showcases the effectiveness of proactive measures like adaptable production systems, diversified trade partners, and robust cybersecurity infrastructure in mitigating the adverse impacts of cyberattacks. Incorporating cyber resilience significantly dampens the reported negative consequences, highlighting the critical role of preparedness in combating digital warfare.

This study underscores the urgent need for a global paradigm shift toward cyber resilience. Collective efforts to bolster cybersecurity infrastructures, foster international cooperation in threat intelligence, and establish open and resilient trade frameworks are crucial in navigating the treacherous labyrinth of AI‐driven cyberattacks. By embracing resilience strategies and fostering global collaboration, we can pave the way for a more secure and prosperous digital future, where interconnectedness becomes a tool for progress, not a vulnerability to be exploited.

## 1 INTRODUCTION

The intricate web of global trade, woven with interconnected networks and digital arteries, pulsates with economic dynamism, although this very interconnectedness harbors a chilling vulnerability: Artificial intelligence (AI)‐driven cyber threats. These advanced threats, transcending mere data breaches, now target critical infrastructure, wielding the potential to sever vital trade routes and inflict profound economic wounds.

Recent history paints a grim picture of this unfolding reality. The 2020 SolarWinds hack, employing sophisticated AI algorithms, compromised the networks of 18,000 organizations, including tech giants (such as Microsoft and FireEye) and government agencies, highlighting the reach and sophistication of AI‐driven cyber threats (Grubbs et al., 2021). Just three years earlier, the NotPetya ransomware attack, fueled by AI‐enhanced propagation techniques, crippled the global shipping industry, inflicting over $10 billion in losses (Bateman, 2020). Its tentacles ensnared major transportation companies, paralyzing their systems and disrupting supply chains with chilling precision (Lika et al., 2018). The ensuing ripple effects, extending far beyond delayed deliveries, triggered economic shocks across nations. The United States, the United Kingdom, Germany, France, China, and India are among the countries most severely affected by the supply chain disruption (Crosignani et al., 2023).

These are not isolated incidents but rather ominous harbingers of a future where AI empowers cyberattacks to pose unprecedented risks to global trade. Their consequences transcend mere inconveniences. AI‐driven cyberattacks can cripple supply chains, leading to shortages, price hikes, and business closures (Corbet & Gurdgiev, 2020). They can erode consumer confidence, impacting brand reputation, inflating unemployment, and stifling economic growth. In extreme scenarios, they can even trigger global recessions (Goodell & Corbet, 2023).

This necessitates a deeper understanding of the intricate interplay between AI‐driven cyber threats, global trade, and economic stability, and underscores the urgent need for comprehensive strategies to enhance cyber resilience and mitigate the far‐reaching consequences of AI‐driven cyber threats on the interconnected global trade ecosystem. Our paper delves into this critical nexus, utilizing a Computable General Equilibrium (CGE) model to analyze the cascading effects of cyberattacks on trade route disruptions and their macroeconomic implications. By simulating these disruptions across interconnected economies and sectors, we aim to illuminate the multifaceted vulnerabilities of global trade in the face of AI‐driven cyber threats.

Furthermore, we evaluate the efficacy of diverse cyber‐resilience strategies, potentially equipping policymakers with invaluable insights to fortify the arteries of the global economy. This research embarks on a critical mission: to navigate the complex landscape of AI‐driven cyber threats, global trade, and economic stability. By employing a CGE model, it unveils the vulnerabilities inherent in our increasingly digitized trade networks, charts a course toward cyber resilience, and ultimately seeks to safeguard the interconnected lifeblood of our globalized world.

## 2 UNVEILING THE SCARS: THE MULTIFACETED IMPACTS OF AI‐DRIVEN CYBERATTACKS

The evolution of AI has significantly transformed the landscape of cyber threats, marking a new era characterized by highly sophisticated and adaptive attacks. AI‐driven cyberattacks refer to cyberattacks that utilize AI and machine learning (ML) algorithms to enhance their effectiveness, stealthiness, and adaptability. These attacks leverage AI capabilities to automate and optimize various stages of the attack lifecycle, from reconnaissance and infiltration to evasion and exploitation. Unlike traditional cyberattacks, which are typically manual or scripted, AI‐driven cyberattacks can autonomously learn and evolve their tactics, techniques, and procedures based on real‐time feedback and environmental changes. These AI‐driven cyberattacks leverage ML algorithms and automation to orchestrate multifaceted assaults on critical infrastructure, autonomously identify vulnerabilities, launch targeted assaults, and rapidly evolve to counteract defense mechanisms, posing significant threats to global trade and economic stability (Blessing, et al., 2022).

The impact of AI‐driven cyberattacks on global trade and economies is multifaceted and pervasive. Recent statistics underscore the severity of these threats. According to the IBM Cost of a Data Breach Report (IBM, 2021), the average cost of a data breach globally reached $4.24 million in 2021, showcasing the financial implications of cyberattacks. Furthermore, the World Economic Forum's Global Risks Report 2022 identified cyber threats among the top risks concerning businesses and economies worldwide (WEF, The Global Risks Report, 2022).

To fully comprehend the scope of these threats, this section dissects the impacts of the most common types of AI‐driven cyberattacks. It categorizes the scars they leave and delves into the intricate consequences these attacks bring upon the delicate flow of international trade. In Table 1, we outline several AI‐driven cyberattacks, detailing their definitions and economic impacts. These attacks exploit AI algorithms to craft deceptive inputs, automate social engineering techniques, enhance malware capabilities, and orchestrate large‐scale disruptions. The economic repercussions of these attacks extend across multiple dimensions, affecting trade, market stability, supply chains, and consumer confidence. Understanding the nature of these threats and their economic ramifications is crucial for devising effective cybersecurity strategies and safeguarding the resilience of the global economy.

**TABLE 1:** AI‐driven cyberattacks.

Types and definitions	Economic impacts
Adversarial attacks: Crafting inputs to deceive ML models.	▪ Disruption of trading algorithms, market volatility, and financial losses. ▪ Incorrect tariff classifications affecting trade agreements, leading to delays or penalties in international trade. ▪ Decreased confidence in AI technologies, hindering economic activities (e.g., supply chain management and financial services). ▪ Ineffective marketing strategies due to misclassification of customer behavior data, reducing market competitiveness. ▪ Overproduction or underutilization of resources impacting supply chain efficiency and economic productivity.
Automated social engineering: Leveraging AI to craft personalized phishing emails, or scams tailored to deceive specific individuals/groups.	▪ Financial losses through fraudulent transactions and unauthorized access to financial accounts. ▪ Disruption of supply chain operations and trade transactions. ▪ Undermined trust in digital communication channels and online transactions, hindering digital trade facilitation initiatives. ▪ Reputational damage impacting competitiveness and trade relationships in global markets.
AI‐enhanced malware: Malicious software with AI capabilities like evasion and polymorphism.	▪ Delays in production, distribution, and delivery of goods, impacting global trade volumes and supply chain efficiency. ▪ Diminished competitiveness due to theft of proprietary information. ▪ Loss of customer trust and confidence, negatively impacting business relationships and market reputation. ▪ Significant costs for cybersecurity measures, diverting funds from core economic activities.
Generative adversarial networks (GANs) attacks: Using GANs to generate synthetic content for disinformation campaigns, financial scams, and intellectual property theft.	▪ Impact on public opinion and societal stability due to disinformation campaigns. ▪ Financial losses from counterfeit goods, financial scams, and market uncertainty. ▪ Impersonation of individuals, damaging reputations, and violating privacy. ▪ Disruption of operations, decreased market competitiveness, and impact on trade relations. ▪ Challenges for regulation, cybersecurity risks, and erosion of trust in digital media.
AI‐powered data poisoning attacks: Injecting malicious data into training data sets to manipulate AI models.	▪ Reduced accuracy and reliability of AI models, impacting financial, and trade‐related decisions. ▪ Financial losses from suboptimal investment decisions, pricing errors, and inefficient resource allocation. ▪ Decreased trust in AI systems and algorithms, hindering their adoption for trade‐related tasks. ▪ Market distortions such as unfair competition and discriminatory practices due to biases in AI models.
Model inversion attacks: Exploiting the transparency of ML models to infer sensitive information about training data or inputs based on the model's outputs.	▪ Risk of privacy breaches in trade‐related data and unauthorized access to confidential information. ▪ Economic sabotage through industrial espionage, compromising competitive advantages, and fair trade practices. ▪ Erosion of trust in AI systems for trade‐related decision‐making, leading to regulatory consequences and financial losses. ▪ Direct financial losses, recovery expenses, and long‐term damage to affected industries and economies.
AI‐driven ransomware: Deployment of AI algorithms to identify and target high‐value data or systems for encryption in ransomware attacks.	▪ Higher ransom demands exacerbating financial losses. ▪ Prolonged recovery process due to AI‐enabled encryption techniques. ▪ Disruption of business operations, leading to downtime, loss of productivity, and financial setbacks. ▪ Erosion of trust in digital systems and confidence in online transactions, affecting consumer behavior and trade relationships. ▪ Introduction of stricter regulations to address ransomware threats, impacting trade policies and business operations.
AI‐powered reconnaissance: Automated gathering and analysis of information about target systems and identifying their potential vulnerabilities (e.g., trade and supply chain networks).	▪ Heightened market instability as attackers gain insights into trade and supply chains, potentially causing fluctuations in prices and demand. ▪ Enable threat actors to identify vulnerabilities and exploit weaknesses in trade networks, undermining the competitive advantage, and causing production delays and shortages. ▪ Stealing intellectual property related to trade secrets, product designs, or market strategies, leading to economic losses and diminished innovation capabilities. ▪ Breaches resulting from reconnaissance attacks can erode trust between trading partners, hindering collaboration, and impeding the formation of new trade agreements and partnerships. ▪ Leading to compliance burdens and increased operating costs. ▪ Disrupt the flow of goods and services, leading to production delays, inventory shortages, and increased logistics costs. ▪ Reputational damage, resulting in a loss of market share as consumers and trading partners seek more secure alternatives.
AI‐driven evasion: Techniques that dynamically adjust attack strategies based on real‐time feedback and environmental changes to evade detection by security systems.	▪ Disruption of trade operations, leading to delays and increased costs. ▪ Compromised trade security, risking fraud, and cyberattacks. ▪ Decreased trust in trade systems, hindering collaboration. ▪ Market uncertainty and volatility due to disrupted trade flows. ▪ Regulatory compliance challenges and legal liabilities. ▪ Loss of competitive advantage, affecting market share and revenue growth.
AI‐powered DDoS attacks: Utilization of AI to orchestrate distributed denial‐of‐service attacks with greater efficiency.	▪ Attacks on e‐commerce platforms and financial institutions cause delays in transactions and shipments, affecting international trade. ▪ Downtime and operational costs from attacks reduce profitability and investment in trade. ▪ Disruption of online services, affecting trade relationships. ▪ Attacks prompt regulatory interventions, increasing compliance costs and affecting trade competitiveness. ▪ Attacks deter investment in digital technologies critical for global trade, hindering progress.
Scalable threats: Utilization of AI to orchestrate large‐scale, coordinated attacks across multiple targets simultaneously.	▪ Physical damage to infrastructure, safety risks to individuals, and disruption of essential services. ▪ Economic losses from service disruptions, operational downtime, and damaged reputation. ▪ Amplified impact of cyberattacks, as these coordinated attacks magnify financial losses and operational disruptions. ▪ Large‐scale attacks overwhelm cybersecurity defenses. ▪ Critical nodes in supply chains suffer disruptions, leading to delays and increased costs.

*Source*: Authors' compilation from the following studies: Malatji and Tolah (2024), Guembe et al. (2022), Yamin et al. (2021), and Sarker et al. (2021).

### 2.1 Disrupted trade operations and supply chains

Malicious actors targeting AI systems in supply chain management, through poisoning models or botnet intrusions, compromise the integrity of trade deals, leading to disruptions in logistics, procurement, and inventory management. An adversarial attack manipulating an AI‐based inventory management system might result in inaccurate stock predictions, creating bottlenecks in the global supply chain (Anbumozhi et al., 2020; Pandey et al., 2020).

### 2.2 Impact on seamless flow of goods and services

Breaches in interconnected systems can result in delays, shortages, or overstocking, disrupting global trade routes. A cyberattack compromising a key logistics system might delay shipments, impacting the timely delivery of goods to international markets, causing financial losses for businesses, and affecting consumer demand (Katsaliaki et al., 2022; Smith et al., 2023).

### 2.3 Operational standstills and reduced productivity

Successful botnet attacks targeting AI‐driven systems used in global trade lead to significant operational downtime. This disruption halts or slows down critical trade functions, including supply chain management, inventory tracking, procurement, and financial transactions. The resultant downtime leads to inefficiencies and delays in executing trade operations. Systems essential for decision‐making, logistics planning, or transaction processing become compromised, causing interruptions in the flow of goods and services across borders. AI‐driven systems affected by cyberattacks may lead to compromised decision‐making processes. As a consequence, businesses may struggle with inaccurate data, flawed predictions, or halted automation, impacting the accuracy and efficiency of trade decisions (El‐Gendy et al., 2023).

### 2.4 Financial losses and long‐term repercussions

Compromised AI models due to adversarial attacks directly translate into financial losses for businesses involved in global trade, through fraudulent activities like misleading predictions or fraudulent fund transfers. This might also ripple into long‐term financial repercussions for affected entities due to factors like unsold inventory or reduced demand. A successful AI‐driven cyberattack altering trade predictive models might cause businesses to invest in incorrect markets or overproduce goods, resulting in financial losses due to unsold inventory or reduced demand (Rosenberg et al., 2021).

### 2.5 Misguided investments and economic setbacks

Incorrect predictions, compromised decision‐making, and flawed strategies due to AI system compromises can lead to misguided investments, pricing errors, and economic setbacks for businesses engaged in global trade. A cyberattack on AI systems that serve trade processes might result in flawed market analysis, leading to a misallocation of resources in new markets, impacting revenue generation and causing economic setbacks (Burri, 2021).

### 2.6 Reputational damage and credibility erosion

Successful cyberattacks on AI‐driven trade systems erode trust among stakeholders, customers, and partners involved in global trade, impacting the reputation and credibility of affected businesses or countries. A breach in the AI system of a multinational trade corporation might lead to a loss of trust among customers and partners, affecting their willingness to engage in future trade deals (Kannelønning & Katsikas, 2023; Smith et al., 2023). Public disclosure of a cyberattack targeting a government's AI systems may impact its standing in international negotiations and trade agreements (Jones, 2023).

### 2.7 Instability and uncertainty

AI‐driven attacks can introduce uncertainties in trade dynamics, causing sudden shifts in supply chains, market inefficiencies and instabilities, and distorted market analyses, thereby disrupting established trade relations. A successful cyberattack that manipulates a country's trade‐related AI models might cause sudden fluctuations in commodity prices and hence demand for certain goods, impacting trade agreements and disrupting established market trends (Katsaliaki et al., 2022; Yi et al., 2020).

## 3 LITERATURE REVIEW

Measuring economic losses from AI‐driven cyberattacks, akin to other large‐scale disasters, is crucial for informing mitigation and recovery strategies. Existing literature explores various methodologies, including input–output (IO) models, social accounting matrix (SAM) multiplier approach, and CGE models.

IO and SAM multiplier models offer a data‐driven approach to estimate disaster impacts. They capture economic interdependencies through a table representing flows of goods and services. Disasters disrupt these flows, causing direct (e.g., infrastructure damage) and indirect effects (e.g., reduced demand). Studies by Okuyama and Santos (2014) and Lenzen et al. (2004) demonstrate their effectiveness in quantifying disaster losses. However, IO models have limitations. They assume fixed production coefficients and linear relationships, neglecting potential price adjustments and behavioral changes. These would potentially lead to overestimated effects of large disruptions. Additionally, IO models often lack the capacity to capture long‐term effects.

Although studies like Eling et al. (2023) specify cyberattacks with IO models as inoperability and economic damage of the targeted sectors, they often neglect broader economic impacts and downstream disruptions. Dreyer et al. (2018) and Kokaji and Goto (2022) address this gap by capturing upstream supply chain disruptions, but downstream impacts remain understudied.

CGE models offer a more advanced and theoretically grounded framework for analyzing disaster and cyberattack impacts. Pioneering work by Rose and Guha (2004) paved the way for their application in cyber threats. Rose (2019) highlights their ability to identify vulnerable sectors and assess economic impacts on growth and employment. Athukorala et al. (2018) further emphasize their strength in capturing interdependencies within global trade networks, crucial for understanding the ripple effects of cyberattacks.

CGE models incorporate nonlinear relationships, price flexibility, substitution possibilities, and endogenous determination of production and consumption patterns. This allows them to capture complex economic responses beyond production, including price adjustments, resource reallocation, consumer behavior, and government intervention (Rose & Liao, 2005). Additionally, CGE models handle supply constraints, making them suitable for analyzing long‐term and dynamic impacts (Wing et al., 2015).

Research acknowledges the importance of resilience in mitigating the economic consequences of cyberattacks, but specific mechanisms and their effectiveness are still being explored. Rose (2019) differentiates between static (maintaining system functionality) and dynamic resilience (recovery speed and intensity). Rose et al. (2019) incorporate various resilience tactics into CGE models (e.g., input and import substitution) to assess their effectiveness in reducing business interruption losses. Additionally, Gertz et al. (2019) specify resilience and recovery in the aftermath of disrupting events through production recapture, reconstruction, and growth dynamics. Rose and Miller (2021) encompass investments in cybersecurity, information sharing, and robust contingency plans. Rose and Chen (2020) paint a vivid picture of potential losses depending on attack severity, repair duration, and response effectiveness. Their work underscores the critical role of technological advancement in cyber resilience.

Although CGE models offer valuable insights, they are computationally complex and require extensive data with strong assumptions about optimizing behavior, limiting their applicability and transparency. Due to their complexities, most existing CGE models are either single‐country or subregional models, neglecting the global nature of cyberattacks. This highlights the need for multiregional models that capture both local and global consequences, particularly for large‐scale AI‐driven cyberattacks, as emphasized by Zhou and Chen (2020).

The limited availability of reliable and comprehensive data remains a major challenge in assessing the economic impacts of cyberattacks. Our study addresses this gap by utilizing the Global Trade Analysis Project (GTAP) global database and a comprehensive CGE modeling framework to examine the ripple effects of large‐scale AI‐driven cyberattacks through global supply chain and trade networks, capturing both upstream and downstream systemic impacts. By harnessing the analytical power of a multiregion CGE model, we aim to illuminate the nuanced economic impacts of these emerging threats and shed light on the most effective resilient strategies for safeguarding the global economy.

## 4 METHODOLOGY

This paper utilizes CGE modeling to investigate the cascading consequences of cyber threats on global trade and supply chain disruptions, while also assessing the effectiveness of implemented cyber‐resilience strategies. We leverage the multiregion, multisector Standard GTAP Model (Version 7), calibrated to the GTAP10 data set on the global economy with a 2014 reference year (Aguiar et al., 2019). This global model offers a detailed portrayal of economic interdependencies across 13 regions and 17 sectors, encompassing 5 production factors (Table 2).

**TABLE 2:** GTAP10 disaggregation.

Sectors		Regions	
1	Agriculture	1	USA
2	Oil and gas	2	UK
3	Mining and extraction	3	EU
4	Processed food	4	China
5	Light manufacturing	5	Japan
6	Heavy manufacturing	6	India
7	Electricity	7	South Korea
8	Gas manufacture and distribution	8	Russia
9	Other utilities and construction	9	North and Latin America
10	Trade	10	Rest of Asia
11	Sea transport	11	MENA
12	Air transport	12	Sub‐Saharan Africa (SSA)
13	Other transport	13	Rest of the World (ROW)
14	Communication	**Production Factors**	
15	Financial services	1	Land
16	Business services	2	Unskilled labor
17	Other services	3	Skilled labor
		4	Capital
		5	Natural resources

*Source*: Authors' aggregation of the GTAP10 database.

### 4.1 Region selection and rationale

The GTAP database integrates detailed bilateral trade, transport, and protection data on regional linkages with individual country I‐O tables, capturing intersectoral linkages within regions, and resembling a global SAM.

The chosen regional aggregation prioritizes major players within the global trade and supply chain network (e.g., the United States and China). This design aims to represent countries with robust supply chain systems that significantly contribute to international trade fluidity. For instance, South Korea, a global leader in technological advancement, exerts substantial influence on supply chains for semiconductors, electronics, and automobiles. Similarly, India's rapidly expanding market, particularly in electronics, software, and services, generates significant demand for resources, raw materials, and finished goods, impacting the global supply chain landscape.

Additionally, the EU and United Kingdom play pivotal roles, especially in areas like automobiles, electronics, and machinery (Germany); luxury goods, automobiles, electronics, and aerospace (France); and financial services (UK). Their well‐developed and efficient supply chains significantly contribute to smooth international trade operations.

### 4.2 Sectoral focus and justification

The sector aggregation scheme emphasizes industries susceptible to immediate cyberattack impacts and those with significant influence on global trade and supply chain flows. For example, the trade and transport sectors heavily rely on digital systems for communication, navigation aviation operations, and logistics management. As Section 2 explains, cyberattacks targeting these systems can disrupt trade transactions, delay shipments, cause accidents, and result in cargo losses, incurring substantial financial losses for businesses. The cyberattacks on Maersk (2017) and the port of Rotterdam (2018) exemplify such disruptions, hindering cargo handling and impacting global trade.

Oil and natural gas are increasingly targeted by cyberattacks (e.g., the 2012 attack on Saudi Aramco impacting crude oil and natural gas production and distribution), which can trigger significant spill‐over effects on other production and trade sectors (Kokaji & Goto, 2022), as well as financial markets. Furthermore, electricity, gas, water, and other utilities play a vital role in cyber‐resilience measures.

Communication networks are crucial for coordinating trade and supply chain activities, and cyberattacks disrupting these networks can hinder collaboration and lead to substantial delays and disruptions. The financial services industry (and to a lesser extent other services) is a key player in the global trade and supply chain, making it exceptionally vulnerable to cyberattacks, with annual costs exceeding $18 million in 2018 (WEF, The Global Risk Report, 2020).

### 4.3 The GTAP model

The GTAP model is a comparative static global model for the world economy. It assumes perfect competition and constant returns to scale (Corong et al., 2017).

**FIGURE 1:**
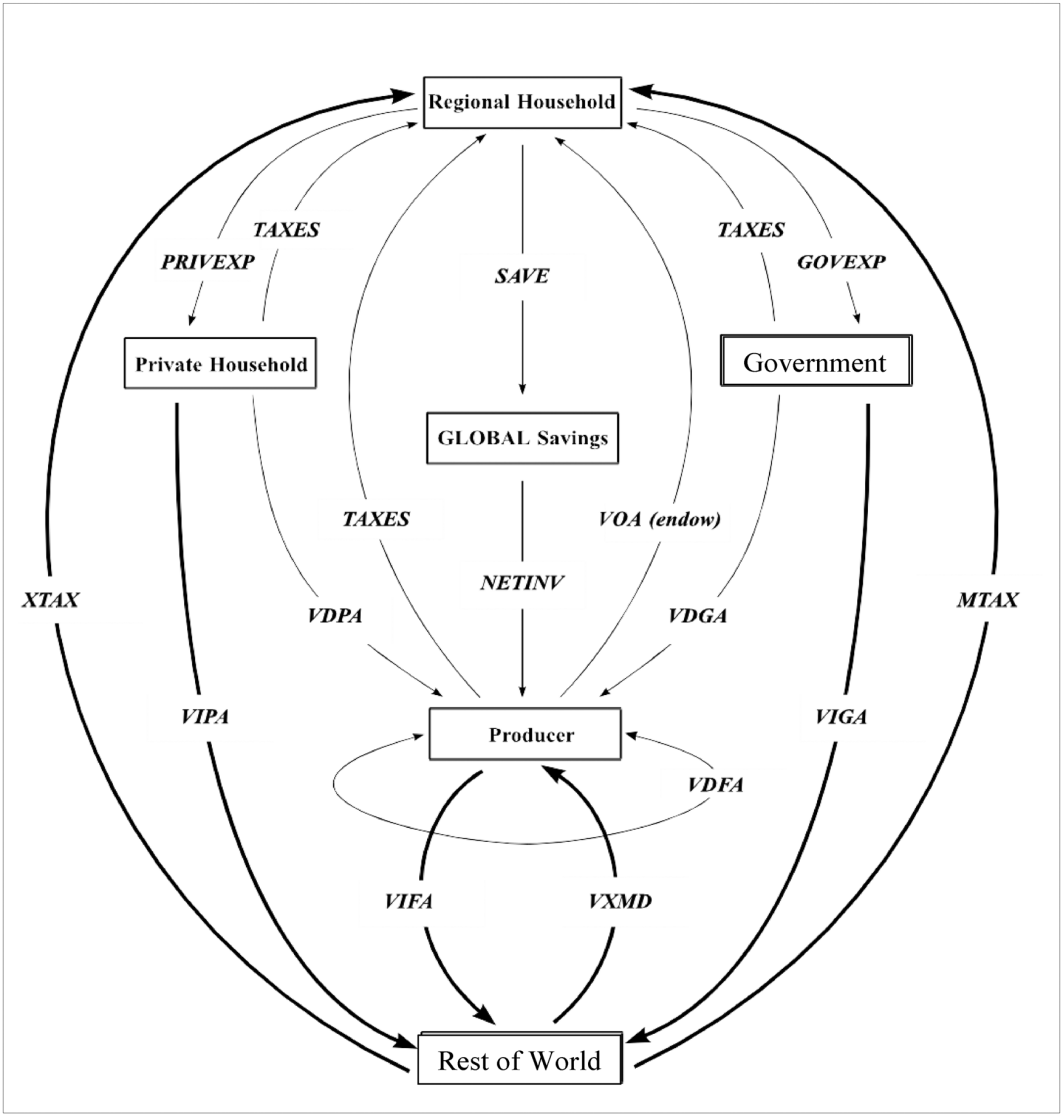
Graphical representation for the GTAP model. *Note*: *VOA (endow)* value added received by the private household in return for the use of its endowments; *XTAX* export taxes; *VDPA* domestic purchases by the private household at agents' prices; *VDGA* domestic purchases by the government at agents' prices; *MTAX* import taxes; *VIPA* imported purchases by private household at agents' prices; *VIGA* imported purchases by government at agents' prices; *VDFA* domestic purchases by firms at agents’ prices; *VIFA* imported intermediate inputs purchases by firm at agents’ prices; *VXMD* exports of commodity I from region r to region s valued at exporter's domestic price. *Source*: Brockmeier (2001).

Figure 1 provides a graphical representation of the model. An aggregate regional consumer follows the Cobb–Douglas utility function. It collects the region's income and allocates between private expenditure, government expenditures, and savings according to constant shares.

**FIGURE 2:**
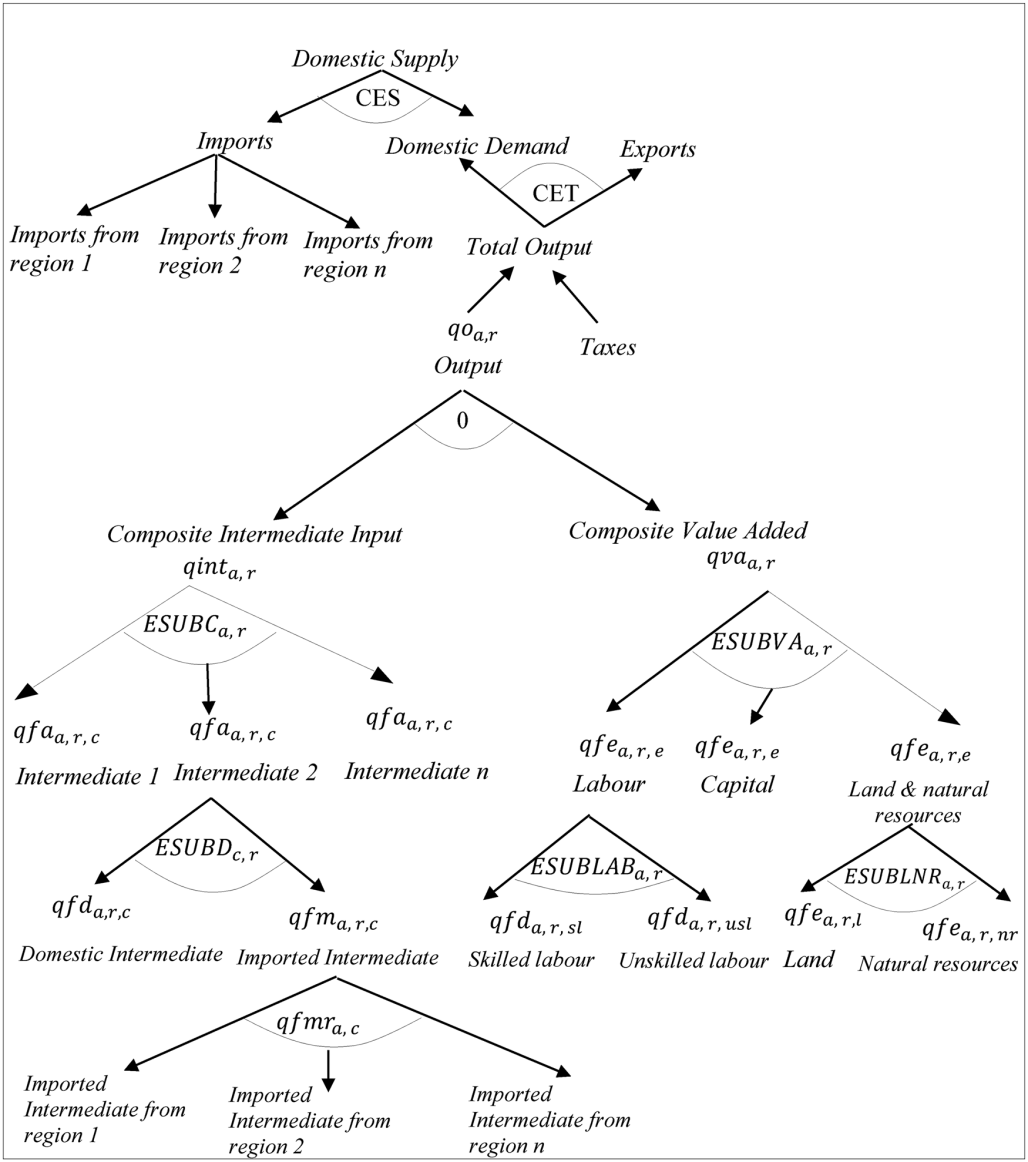
Production and trade flows in the GTAP model. *Source*: Authors' elaboration.

The model uses a sequence of nested Constant Elasticity of Substitution (CES) production functions to specify activity behavior (Figure 2). Each activity combines aggregate value added and aggregate intermediate inputs using Leontief coefficients. The optimal bundle of production factors is governed by a CES function (from capital, composite labor, and composite land) with region‐ and activity‐specific substitution elasticities. The aggregate labor is defined by a CES function over two arguments—skilled and unskilled labor. CES production technologies define aggregate intermediate input from individual intermediate inputs. Each individual input is a CES aggregate of imported and domestic intermediate components. Similarly, the model adopts the Armington function for trade: two‐level nested CES import functions and two‐level nests of constant elasticity of transformation (CET) export functions.

### 4.4 AI‐driven cyberattacks in a general equilibrium framework

This global multisector multiregion CGE model serves as a robust instrument illustrating the interconnectedness of diverse sectors within and across economies. Based on the I‐O structure of each country, the model ties all countries together through a complex web of direct and indirect trade connections and supply chain interactions, establishing a general equilibrium setting wherein prices and quantities are determined simultaneously clearing primary factor markets and domestic and international commodity markets.

This is particularly relevant to AI‐driven cyberattacks, because their impacts are not confined to the immediate sector/country of concern, but they spread across the globe, as shown in Section 2. When a country (A) is affected by a cyberattack that targets trade or trade‐related service sectors (e.g., transportation, communication, and financial services), it will face a higher price of imported inputs. Prices of final commodities in sectors with high intensity of imported inputs would increase. Demand for imported intermediate inputs would consequently drop and they would be substituted with domestic inputs according to the CES substitutability. In addition, demand for final domestic substitutes would increase. These together would generate cost‐push and demand‐pull increases in prices in sectors with high trade intensity. Production factor markets would experience lower demand.

The ripple impacts are captured throughout the trade network and supply chain. Another country (B), which would have exported these intermediate inputs at a lower price in the absence of the cyberattack, would experience a reduction in demand in product and factor markets. The factor markets clearing conditions in both countries A and B (i.e., fixed supply and full employment) would lower production factor returns. Both countries would suffer a reduction in productivity and inefficient allocation of resources. The more severe the cyberattacks and the more exposed a country is to trade, the greater the losses in productivity and efficiency induced by cyber threats.

### 4.5 Tracking cyberattacks impacts across sectors and regions

This general equilibrium framework not only captures the first‐order effect of a cyberattack but also delves into the second‐order effects that operate through upstream and downstream sectors and regions. Within this framework, the impact of a cybersecurity breach in one sector on other sectors or other regions is determined, *inter alia*, by the level of economic complexity, interconnectedness, and connectivity among sectors and economies (Borg, 2005).

If a cyberattack affects a sector with strong forward linkages, which measure the relative importance of the sector as a supplier of outputs to other sectors in the economy, then we would expect noticeable ripple effects on other sectors and the economy as a whole. For example, the USA 2021 Colonial Pipeline cyberattack had ripple effects on forward‐linked sectors and led to disruptions in transportation (trucking and air travel), increased costs for manufacturers and retailers, and potential power outages due to limited fuel for electricity generation. On the other hand, the pattern of the impacts would be different if the cyberattack targets a sector with strong backward linkages. Backward linkages reflect the economic connection between one sector and other sectors supplying inputs, either directly or indirectly. Hence, the impact would depend mainly on the ability of this sector to recover and maneuver post‐attack.

By the same analogy, the spread of the impact across regions depends on trade and supply chain connectivity. Trade disruptions can be attributed to two effects: output and relative price changes. Output changes reflect downward shifts of the CET export (and the CES import) frontiers due to decreases (and increases) in domestic output. On the other hand, relative price changes reflect movements along the CET export (and the CES import) frontiers due to increases (and decreases) in relative prices of domestic commodities and their foreign substitutes. Indeed, the complex tapestry of trade and supply chain connectivity served as a conduit for the 2017 NotPetya cyberattack, weaving a web of disruption that ensnared not only Ukraine but also the United States and Russia. In 2017, the United States was Ukraine's fourth‐largest export market, and trade between Russia and Ukraine remained significant. This intertwined trade embodies US businesses’ reliance on Ukrainian inputs and exposed Russian industries to supply chain disruptions caused by NotPetya's attack.

## 5 SIMULATION SCENARIOS

Based on Section 2, we designed three hypothetical simulation scenarios to elucidate the cascading impacts of AI‐driven cyberattacks and cyber resilience on trade flows, economic structure, and productivity across regions. Each scenario includes three types of shocks (namely, trade, supply, and productivity) that mimic the disruptive effects of such attacks on various facets of the global economy. Furthermore, these scenarios encompass the implementation of four cyber‐resilience measures to assess their effectiveness in mitigating the adverse effects of cyberattacks.

### 5.1 Cyberattack shocks

To represent the impact of cyber threats on the model, we introduce the following shocks:

#### 5.1.1 Trade shock

This shock increases trade costs, reflecting the increased complexity and uncertainty associated with international trade in the presence of AI‐driven cyber threats. We introduce an *ad valorem* tariff shock to imports and exports in all goods and services for the main international traders, representing the additional costs and delays due to cyberattacks, and these cyberattack‐targeted countries are the United States, China, the EU, the United Kingdom, Japan, South Korea, India, and Russia.

#### 5.1.2 Supply shock

This shock affects the production costs of selected industries, representing the disruption caused by AI‐driven cyberattacks in the cyberattack‐targeted countries. We employ a multiplicative cost shock to industries that are heavily reliant on digital technologies or interconnected supply chains, and these are agriculture, oil and gas, mining and extraction, processed food, and light and heavy manufacturing.

#### 5.1.3 Productivity shock

In order to signify the tangible losses or impairment of physical assets and infrastructure caused by cyber threats, we introduce multiplicative total factor productivity reductions in the cyberattack‐targeted countries. These are equivalent to sectors becoming less efficient in their use of inputs due to cyber disruptions, and this infrastructure includes electricity, gas manufacture and distribution, other utilities and construction, various transport, communication, financial, business, and other services.

### 5.2 Cyber‐resilience mechanisms

Cyberattacks are dynamic in nature and, hence, it is extremely difficult and costly to mitigate their negative impacts upfront. We thus assume that economies build cyber‐resilience strategies for the “known” threats, in the sense that the system embodies “machine learning” mechanism that allows the system, after exposure to a threat, to learn how to deal with similar incidents.

In the face of cyberattacks, producers can adapt their production and trade behavior in two key ways. First, cyberattacks can disrupt normal operations, forcing producers to find more efficient ways to use resources. This might involve minimizing waste, conserving inputs, and adjusting production processes. These adaptations can be refined and implemented more broadly, leading to improved factor productivity. Second, producers can increase their ability to switch between different resources and suppliers in response to disruptions. This could involve diversifying production inputs and trade partners. By making their systems more adaptable, producers become more resilient to future cyberattacks (Rose & Liao, 2005).

Against this background, we implement four cyber‐resilience mechanisms, which enable the cyberattack‐targeted countries to:

1. Substitute domestic goods with their imported counterparts more easily if any of these became inaccessible after a cyber‐related disruption. The Armington substitution elasticities between imported and domestic goods and between imports from different regions are higher.
2. Divert goods between export markets and domestic markets more easily if any market has decreasing demand, which implies lesser disruption of the product supply in the cyber aftermath. The CET export functions exhibit higher transformation elasticities between domestic and export markets and between different export markets.
3. Diversify their suppliers of intermediate inputs and widen their supply chain. The CES import functions exhibit higher substitution elasticities between domestic and imported inputs and between imported inputs from different trade partners.
4. Substitute between primary and intermediate inputs. The production technology compasses CES (instead of fixed Leontief coefficients) between aggregate value added and aggregate intermediate input. This replicates cases of temporary adjustment made in certain production activities where essential intermediate inputs (e.g., electricity and water) can be replaced by (or can replace) other primary inputs (e.g., labor). A temporary shift from fully automated production processes to less sophisticated mechanical (or even manual) processes, and the opposite shift to automated processes under losses in labor, can help to buffer against the severity of cyberattacks and to reduce the production disruption (e.g., electricity cut induced by cyberattacks).

### 5.3 Cyberattack and cyber‐resilience scenarios

**FIGURE 3:**
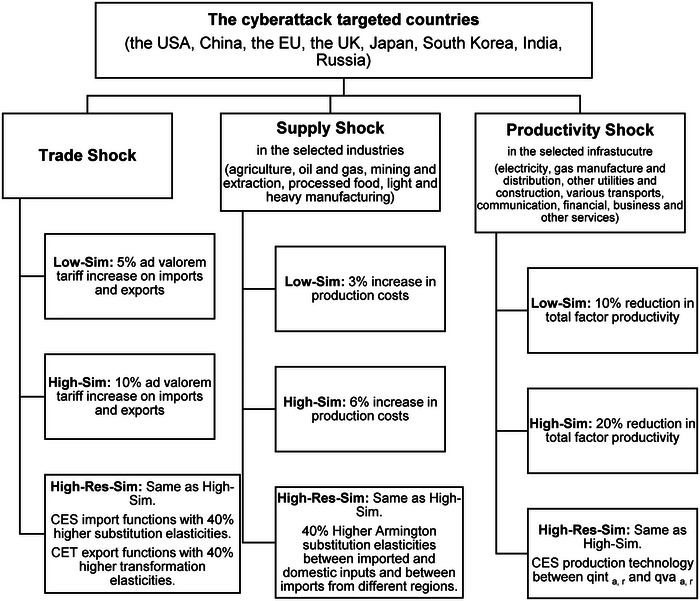
Simulation scenarios. *Source*: Authors' elaboration.

Based on the above two subsections, we design three simulation scenarios, encompassing the three types of cyberattack shocks as well as four cyber‐resilience mechanisms, as presented in Figure 3.

#### 5.3.1 Low‐sim (Low‐level cyberattack scenario)

This scenario represents a low‐level cyberattack, with a small number of cybersecurity breaches. The number of cybersecurity breaches in this scenario is 50% of the number of breaches in the high‐level cyberattack scenario. This scenario includes an increase in trade costs by 5% for the cyberattack‐targeted countries, an increase in production costs by 3% in the selected industries, and a reduction in productivity by 10% in the selected infrastructure sectors.

#### 5.3.2 High‐Sim (High‐level cyberattack scenario)

This scenario represents a high‐level cyberattack, with a large number of cybersecurity breaches. The number of cybersecurity breaches in this scenario is twice the number of breaches in the low‐level cyberattack scenario. This scenario includes: an increase in trade costs by 10% for the cyberattack‐targeted countries, an increase in production costs by 6% in the selected industries, and a reduction in productivity by 20% in the selected infrastructure sectors.

#### 5.3.3 High‐Res‐Sim (High‐level cyberattack with cyber‐resilience scenario)

This scenario represents the same number of cybersecurity breaches as in the high‐level cyber threat scenario, and it incorporates the four resilience mechanisms for the cyberattack‐targeted countries. These resilience mechanisms are higher import substitution elasticities, higher export transformation elasticities, higher substitution elasticities between imported and domestic inputs, and CES production technology for value added and intermediate demand. The higher values of various elasticities are implemented through a multiplicative increase of 1.4, keeping the heterogeneity of elasticity values across sectors and regions as the original elasticity values in the GTAP10 database exhibit (Aguiar et al., 2019).

## 6 RESULTS AND ANALYSIS

The simulations show that cyber threats can have a significant negative impact on global trade, with the largest impact on economies that are more reliant on digital technologies and interconnected supply chains. However, cyber‐resilience measures can help to mitigate these risks, the High‐Res‐Sim scenario exhibits mitigating negative impacts, indicating the potential of cyber‐resilience strategies in cushioning the effects of cyber threats on international trade.

### 6.1 Changes in real GDP

The simulation results depict varying degrees of negative impacts on the real GDP of major regions under different cyber threat scenarios in Table 3. For the low‐level cyber threat, the decrease in real GDP remains relatively minor, ranging from 0.02% to 0.25%. These modest reductions reflect the impact of a low‐level cyber threat, with limited breaches. Countries such as China, Japan, and South Korea experience slightly higher declines in real GDP. The three economies account for around 20% of the world trade, and they are major mutual trading partners with strong intra‐industry trade in manufacturing products reflecting China's industrial structure and advanced technology. This suggests a marginally more pronounced effect in economies with high economic exposure and strong trade interconnectivity.

**TABLE 3:** Changes in real GDP (%).

Regions	Low‐Sim	High‐Sim	High‐Res‐Sim
USA	−0.05	−1.40	−0.70
UK	−0.02	−1.20	−0.60
EU	−0.05	−1.35	−0.75
China	−0.15	−2.20	−1.60
Japan	−0.10	−2.00	−1.40
India	−0.02	−1.15	−0.85
South Korea	−0.10	−1.95	−1.70
Russia	−0.05	−1.25	−0.95
North and Latin America	−0.25	−2.40	−2.00
Rest of Asia	−0.10	−2.00	−1.60
MENA	−0.05	−1.95	−1.60
SSA	−0.20	−2.40	−2.00
ROW	−0.15	−2.25	−1.85

*Source*: Authors' elaboration on model results.

Notably, the High‐Sim scenario reveals more substantial declines in real GDP across all regions. The United States, the United Kingdom, the EU, China, Japan, and India encounter more pronounced reductions. This demonstrates the heightened detrimental impact of a high‐level cyber threat, resulting in more significant economic disruptions.

Interestingly, the most affected regions are North and Latin America and sub‐Saharan Africa (SSA). The two regions are heavily dependent on imported intermediate inputs essential for their domestic production, and the bulk of these inputs are imported from the most affected countries (e.g., China, Japan, India, South Korea, and the United States), which explains the manifold impact. Cyberattacks that target ports, shipping companies, and other logistics providers would severely disrupt these intermediate goods' flow, and cause severe supply chain disruptions.

Cyber resilience can significantly mitigate the negative impact of cyber threats. Under the High‐Res‐Sim scenario, the United States, the United Kingdom, the EU, China, and Japan experience a sizable difference compared with real GDP reduction under the High‐Sim scenario, whereas results for India, South Korea, and Russia under the two scenarios are not significantly different.

### 6.2 Trade prices and volumes

All regions experience deterioration in terms of trade (ToT), meaning their export prices rise less than their import prices (Table 4). Sharp ToT declines occur in countries with high dependency on digital technologies and strong interconnected supply chains (e.g., the United States) and countries that experience increases in production cost and import prices while their exports face muted price increases due to dampened global demand (e.g., China).

**TABLE 4:** Changes in terms of trade (ToT) (%).

Regions	Low‐Sim	High‐Sim	High‐Res‐Sim
USA	−1.20	−2.30	−1.8
UK	−0.90	−1.80	−1.5
EU	−0.70	−1.00	−0.8
China	−2.50	−4.40	−3.2
Japan	−0.60	−1.20	−1.0
India	−0.40	−0.90	−0.7
South Korea	−0.60	−1.30	−1.1
Russia	−0.40	−0.80	−0.6
North and Latin America	−0.70	−1.60	−1.5
Rest of Asia	−0.40	−1.40	−1.2
MENA	−0.30	−1.40	−1.2
SSA	−0.70	−1.60	−1.5
ROW	−0.60	−1.50	−1.4

*Source*: Authors' elaboration on model results.

Economies that are more reliant on exports experience higher ToT deteriorations (e.g., China, Japan, and South Korea) compared with other economies like the United Kingdom, India, and Russia. Moreover, the balance between export competitiveness and vulnerability to import price increases (in countries like India and South Korea) determines the extent of the ToT impact.

Other regions that are less digitized and less dependent on international trade (e.g., SSA) are yet not immune to impacts, as disruptions in global supply chains, increased trade costs, and global economic slowdown can still affect their import prices. The cyber‐resilience scenario mitigates ToT declines by allowing countries to substitute domestic inputs for disrupted imports; however, these positive effects are trivial, as this higher substitutability might come at the expense of higher domestic production costs and reduced overall economic efficiency.

A severe cyberattack can disrupt production, damage infrastructure, and erode consumer confidence, leading to a decline in exports for regions that are heavily reliant on manufacturing, technology, or global supply chains. Indeed, China, the United States, the United Kingdom, and the EU experience the sharpest export declines in the High‐Cyber Threat scenario due to their heavy reliance on digital technologies and interconnected supply chains.

The disruptive impact of this cyberattack on global trade flows is shown by the sharp reductions in China exports (8.2%) and US exports (5.6%) (Table 5). Chinese exports heavily rely on the American markets, whereas US export markets are more diversified. At the baseline scenario, the United States absorbs around 20% of total Chinese exports, whereas China contributes 7% of total US exports. Therefore, cyberattacks would generate higher declines in Chinese exports compared to those of the US's exports; the latter will still decline as higher production costs reduce the US products’ competitive advantages in the global markets. Under the High‐Cyber Threat scenario, China experienced a sharp decrease in exports, the sector that counts for more than one‐fifth of GDP, and this contributed to the country's overall macroeconomic loss, whereas the US exports experienced lesser drops.

**TABLE 5:** Changes in trade volume (%).

Regions	Low‐Sim		High‐Sim		High‐Res‐Sim	
	Export	Import	Export	Import	Export	Import
USA	−3.70	−4.14	−5.60	−7.60	−4.30	−6.10
UK	−2.40	1.53	−3.70	0.72	−2.00	1.20
EU	−1.90	0.90	−2.50	1.10	−2.00	−0.20
China	−4.50	−6.29	−8.20	−9.47	−6.50	−7.30
Japan	−0.90	−1.25	−1.30	−3.60	−1.00	1.80
India	−0.70	−2.22	−1.00	4.18	−0.90	0.80
South Korea	−1.10	0.80	−1.90	−1.24	−1.70	1.34
Russia	0.80	2.53	−0.70	0.40	−0.40	0.30
North and Latin America	1.30	1.20	2.70	1.08	2.20	−0.40
Rest of Asia	1.20	2.10	1.80	1.80	1.40	2.10
MENA	0.60	1.70	1.20	−2.85	1.00	2.00
SSA	1.80	2.70	2.22	1.02	2.20	3.00
ROW	1.70	2.30	4.90	2.38	2.40	2.30

*Source*: Authors' elaboration on model results.

These main traders’ import patterns can be mixed, with potential increases in essential imports needed for recovery (the United Kingdom and the EU) or decreases due to lowered confidence and economic activity (the United States and China). Some regions’ recovery plans might necessitate an increase in imports of critical goods or services needed for recovery, such as replacement equipment, emergency supplies, or cybersecurity services. Other countries experience an increase in imports that is attributed to trade diversion, which suggests complex trade dynamics at play and hence the need for detailed bilateral and trade analysis, as explained below.

Comparably, South Korea, Japan, and India show smaller export declines, reflecting their growing reliance on digital infrastructure and trade integration. Their import patterns, which show slight declines or even increases, are determined by import substitution opportunities, trade diversion routes, and their specific needs for economic recovery.

North and Latin America, the Rest of Asia, Middle East and North Africa (MENA), SSA, and the Rest of the World, generally, experience increases in trade due to their lower dependence on digital technologies and international trade as well as trade diversion and creation opportunities created after disruptions in the main trader routes and the necessity for alternative import sources and trade pathways.

The introduction of resilience measures like improved cybersecurity infrastructure and trade facilitation helps moderate the decline in exports and imports compared with the High‐Cyber Threat scenario. This demonstrates the critical role of proactive resilience building in minimizing the economic impacts and trade disruptions of cyberattacks.

Furthermore, diversification of trade partners and reliance on domestic production can also play a role in mitigating the impact and minimizing the need for large‐scale imports after a cyberattack, leading to lower overall trade imbalances. Indeed, the High‐Cyber Threat scenario has trivial impacts on India's trade. The country's “Look East” policy and “Make in India” initiative strengthen its trade ties with East and South East Asian countries, promote domestic manufacturing, and reduce reliance on imports (Roy, 2020).

### 6.3 Trade routes and diversions after cyber threats

This subsection depicts changes in trade routes and structures, explaining potential trade diversions for the major global traders, under the high cyberattack scenario. Overall, major trade partners like China and the United States experience disruptions in their direct exchanges, prompting alternative routes and benefiting intermediary countries like India, Rest of Asia, Japan, and North and Latin America (Table 6).

**TABLE 6:** Changes in trade routes, High‐Res‐Sim (%).

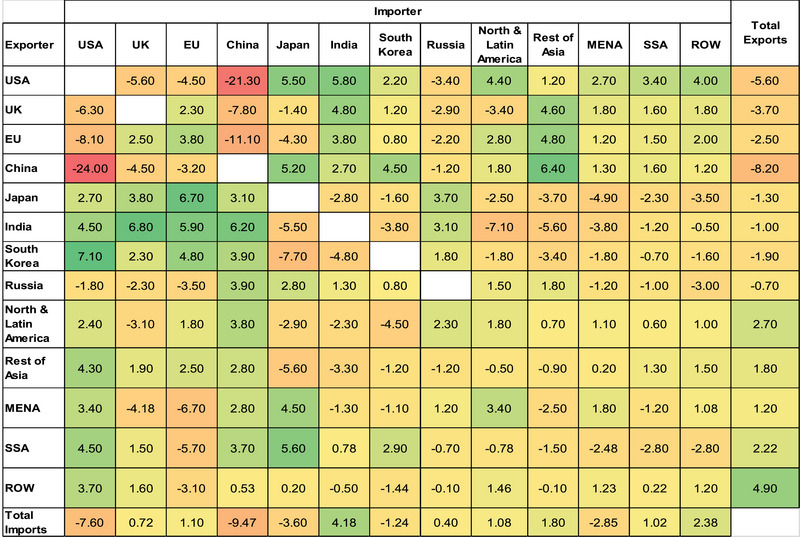

*Note*: The color gradient denotes the magnitude of percentage changes in trade volumes, showcasing the highest percentage increases in green and the highest percentage decreases in red.

*Source*: Authors' elaboration on model results.

Looking at the economic and trade structures for China, the United States, the United Kingdom, and the EU, their exports are likely to be exported to each other's markets through third countries, creating huge flows of trade diversions.

China's exports to the United States dropped sharply by −24% and its exports to the United Kingdom and the EU decreased by −4.5% and −3.2%, respectively. Conversely, the trade diversion effect might lead to increased Chinese exports to other countries outside the United States; mainly the Rest of Asia (6.4%), Japan (5.2%), and South Korea (4.5%), and the three regions' exports to the United States increased by 4.3%, 2.7%, and 7.1%, respectively. This suggests that Chinese exports to the United States have been diverted to other Asian countries before reaching the American markets.

Similarly, US exports to China dropped by 21.3%. The observed increases in US exports to India (5.8%), Japan (5.5%), North and Latin America (4.4%), and South Korea (2.2%) along with surges in their exports to China imply that Chinese imports from the United States have taken diverted routes through third countries.

The examination of trade routes and diversions reveals the complex dynamics of global trade networks in response to cyber disruptions. Major trade partners like China, the United States, the United Kingdom, and the EU may experience shifts in trade flows as a result of disrupted direct exchanges, leading to trade diversions through third countries. The deployment scale of AI technologies in the United States and China amplifies their vulnerability to cyberattacks, resulting in significant disruptions to their trade routes and economic activities. Meanwhile, third countries may capitalize on these disruptions by offering alternative supply routes and trade pathways, thereby mitigating the overall impact on global trade.

### 6.4 Systematic sensitivity analysis

To assess the robustness of our results, we conducted a systematic sensitivity analysis using a Monte Carlo approach (Belgodere, 2011). The systematic sensitivity analysis is conducted for one of the main parameters in the model, namely, the elasticity of substitution between labor and capital. We investigated how varying these elasticities within a specified range influenced the model's outcomes, specifically focusing on percentage changes in export volume for the United States under the Low‐Sim (Low‐level Cyberattack) scenario.

The systematic sensitivity analysis involved 3,000 Monte Carlo simulations, each with a unique set of randomly generated elasticity values for the two production factors. These values were drawn from independent, identically distributed normal distributions. The mean of each distribution was set to the original GTAP elasticity values used in the model, while the variance was defined as half of the mean.

The systematic sensitivity analysis revealed that the percentage changes in export volume for the United States under the Low‐Sim scenario are not dependent on the values of substitution between production factors. The distribution of outcomes was centered around the original elasticity values used in the model.

**FIGURE 4:**
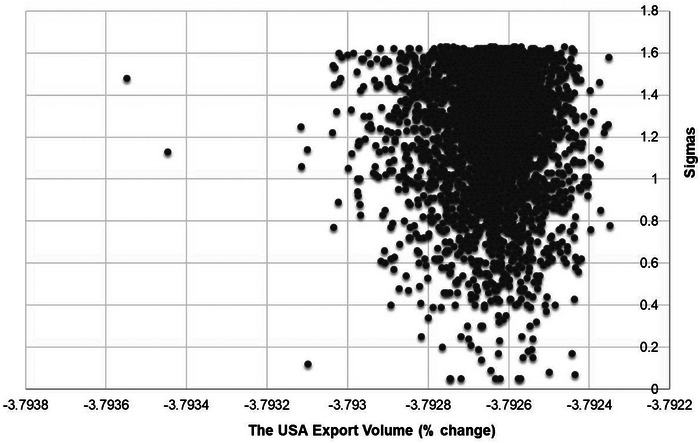
Systematic sensitivity analysis: USA export changes (%), Low‐Sim. *Source*: Authors' elaboration on model results.

The scatter plot presented in Figure 4 visually depicts the relationship between percentage changes in export volume for the United States and the standard deviation (i.e., sigma) of the normal distributions used to generate the elasticity values. It is evident that there is no clear linear relationship, and the general direction and magnitude of the impact on the US exports under the Low‐Sim scenario remain relatively consistent. This suggests that the percentage changes in export volume for the United States are primarily determined by the simulated shocks. The systematic sensitivity analysis demonstrates the robustness of the results to changes in values of the elasticity of substitution between production factors.

## 7 NAVIGATING THE CYBER LABYRINTH: TRADE RESILIENCE IN THE DIGITAL AGE

Our journey through the cyber labyrinth of global trade has illuminated the potential perils, lurking in the shadows of interconnectedness. AI‐driven cyberattacks threaten the very fabric of international trade, unleashing cascading disruptions on economies, supply chains, and trade routes. This study, utilizing the intricate lens of CGE modeling, unveils the nuanced tapestry of these threats, shedding light on their ripple effects across sectors and regions.

The stark picture painted by our simulations underscores the vulnerability of economies heavily reliant on digital technologies and interwoven supply chains. The specter of cyberattacks looms large over real GDP, trade prices, and volumes as well as trade routes, with potentially devastating consequences for economic stability and prosperity. Notably, the deployment scale of AI technologies in countries like China, the United States, the United Kingdom, and the EU exacerbates their susceptibility to cyber threats, leading to more pronounced economic disruptions. Conversely, third countries may benefit from trade diversions and alternative supply routes, mitigating the overall impact on global trade dynamics.

However, amidst the storm clouds of cyber threats, a beacon of hope emerges in the form of cyber resilience. Our findings convincingly demonstrate the potency of proactive measures in staving off the worst impacts of digital warfare. By fostering adaptable production systems, diversifying trade partners, and investing in robust cybersecurity infrastructure, economies can equip themselves to navigate the treacherous landscape of AI‐driven cyberattacks.

The High‐Res‐Sim scenario serves as a testament to the efficacy of resilience strategies. The diminished negative impacts on economies under this scenario highlight the crucial role of preparedness in mitigating the economic carnage wrought by cyber threats. This reinforces the urgent need for a global imperative toward cyber resilience, with a focus on collective efforts to improve cybersecurity infrastructures, foster international cooperation in information sharing and threat intelligence, and promote open and resilient trade frameworks.

In conclusion, the cyber labyrinth presents a formidable challenge to the smooth functioning of global trade. Yet, within this maze lies the potential for a brighter future. By embracing cyber‐resilience strategies, investing in robust digital defenses, and fostering international collaboration, we can pave the way for a more secure and prosperous world, where the flow of trade remains unfettered by the specter of cyber threats. This is the imperative of our time, the key to unlocking a future where interconnectedness becomes a tool for progress, not a vulnerability to be exploited.

## 8 LIMITATIONS AND FUTURE RESEARCH DIRECTIONS

Although this study sheds light on the broader impacts of AI‐driven cyberattacks, it acknowledges the need for a more granular exploration of attack types and their specific consequences. Future research could benefit from developing detailed scenarios specific to various types of AI‐driven cyberattacks and their associated cyber‐resilience approaches. This could involve modeling distinct scenarios for data theft, short‐term denial‐of‐service attacks, and hardware damage, each with tailored resilience strategies. Additionally, modeling different aspects of cyber resilience (e.g., recovery speed, adaptive capacity, and organizational learning) would offer a richer understanding of the overall resilience landscape.

In addition, our model currently does not account for uncertainty and risk‐aversion behavior. Integrating probabilistic elements to reflect the unpredictable nature of cyberattacks and their potential cascading effects, and capturing the risk‐averse decision‐making of economic agents in response to cyber threats would enhance the analysis, as these can influence investment patterns, trade flows, and technology adoption.

Finally, in order to effectively capture the complex dynamics of cyber–economic interactions, future research could utilize a recursive dynamic stochastic CGE approach. This would allow for more accurate modeling of repair and reconstruction efforts, further enhancing the realism and predictive power of the simulations.
